# Ultrasensitive haptoglobin biomarker detection based on amplified chemiluminescence of magnetite nanoparticles

**DOI:** 10.1186/s12951-019-0569-9

**Published:** 2020-01-07

**Authors:** Narsingh R. Nirala, Yifat Harel, Jean-Paul Lellouche, Giorgi Shtenberg

**Affiliations:** 1 Institute of Agricultural Engineering, ARO, The Volcani Center, 50250 Bet Dagan, Israel; 2 Department of Chemistry, Nanomaterials Research Center, Institute of Nanotechnology & Advanced Materials (BINA), Bar-Ilan University, 5290002 Ramat Gan, Israel

**Keywords:** Chemiluminescence, Haptoglobin, Magnetic iron oxide nanoparticles, Mastitis, Pathogens

## Abstract

**Background:**

Haptoglobin is an acute-phase protein used as predicting diagnostic biomarker both in humans (i.e., diabetes, ovarian cancer, some neurological and cardiovascular disorders) and in animals (e.g., bovine mastitis). The latter is a frequent disease of dairy industry with staggering economical losses upon decreased milk production and increased health care costs. Early stage diagnosis of the associated diseases or inflammation onset is almost impossible by conventional analytical manners.

**Results:**

The present study demonstrates a simple, rapid, and cost-effective label-free chemiluminescence bioassay based on magnetite nanoparticles (MNPs) for sensitive detection of haptoglobin by employing the specific interaction of hemoglobin-modified MNPs. The resulting haptoglobin-hemoglobin complex inhibits the peroxidase-like activity of luminol/H_2_O_2_-hemoglobin-MNPs sensing scheme and reduces the chemiluminescence intensities correspondingly to the innate haptoglobin concentrations. Quantitative detection of bovine haptoglobin was obtained within the range of 1 pg mL^−1^ to 1 µg mL^−1^, while presenting 0.89 pg mL^−1^ limit of detection. Moreover, the influence of causative pathogenic bacteria (i.e., *Streptococcus dysgalactiae* and *Escherichia coli*) and somatic cell counts (depicting healthy, sub-clinical and clinical mastitis) on the emitted chemiluminescence radiation were established. The presented bioassay quantitative performances correspond with a standardized assay kit in differentiating dissimilar milk qualities.

**Conclusions:**

Overall, the main advantage of the presented sensing concept is the ability to detect haptoglobin, at clinically relevant concentrations within real milk samples for early bio-diagnostic detection of mastitis and hence adjusting the precise treatment, potentially initiating a positive influence on animals’ individual health and hence on dairy farms economy.

## Background

Acute-phase proteins (APP) are prevalent predicting indicators (biomarkers) for diagnostic health status estimation both in humans and animals, utilizing their distribution alteration in plasma due to infection, inflammation or trauma [1–6]. The acute-phase response of homeostasis disturbance produces a cascade of mRNA upregulation which results in some blood proteins augmentation, while decreasing the synthesis of others [7]. A valid example of this response is the hepatically derived APP, e.g., C-reactive protein, serum amyloid A and haptoglobin (Hp), which are released by the hepatocytes cascade due to cytokine stimulation [5, 8–11]. The latter is a widely used APP biomarker both in humans (i.e., diabetes, ovarian cancer, some neurological and cardiovascular disorders) and in animals (e.g., bovine mastitis), increasing its concentration in plasma up to 100-folds [10]. Hp is widely applied for health status evaluation of dairy cows suffering from bovine mastitis, a recurrent disease of the dairy industry that causes substantial economical loses considering increased health care costs and decreased milk production [1, 5, 6]. Hp is usually secreted by permeable mammary vascular cells, thus augmenting its concentration in milk [12]. The mammary gland inflammation is classified as sub-clinical, clinical, and chronic forms, and is dependent on the causative pathogen origin, animal’s lactation state and immune health [10]. Conventionally, bovine mastitis detection depends on the efficiency of the methods designed to estimate somatic cell counts (SCC), indicate inflammation, identify the causative microorganisms or quantify the biomarkers associated with the disease [6]. Milk and plasma Hp distributions are commonly detected by conventional immunoassays based on hemoglobin (Hb) binding affinity, which are expensive and time-consuming [2, 6].

Chemiluminescence (CL) is a practical opto-chemical approach for designing real-life sensing applications, e.g., safety control within food and pharmaceutical industries, environmental hazardous contaminants monitoring and clinical examination, using the discharged radiation for high sensitivity analysis of the target molecule [13–18]. The addition of superparamagnetism characteristics onto CL bioassays in means of magnetic nanoparticles (MNPs), alleviate the high-throughput immunoassay protocols by convenient separation, widespread the linear sensing range, enhance the reaction kinetics and encompass high surface area for collected biomolecular recognition [19]. Indeed, ample research reports employed MNPs within CL bioassays presenting a list of innovative ultrasensitive sensing schemes [20–25]. Jie et al. have employed gold-coated MNPs for ultrasensitive targeting of fumonisin B1 in cereals and wheat based on CL immunoassay [22]. The developed assay presented a low detection limit of 27 pg mL^−1^ fumonisin B1 and a dynamic range of 0.05 to 25 ng mL^−1^. The high-throughput screening methodology overpowered conventional analytical performances and presented recoveries of 91–110% in real samples [22]. A similar sensing concept was used for zearalenone mycotoxin (produced by Fusarium species) ultrasensitive CL detection [26]. The authors reported a 50-fold pre-concentration of zearalenone in bulk vine samples and its detection as low as 4 pg mL^−1^, more than an order of magnitude increase in sensitivity with respect to colorimetric assay and to MNPs-enzyme-linked immunosorbent assay (ELISA) without pre-concentration procedure (detection limits of 0.06 and 0.1 ng mL^−1^, respectively). Zhang et al. have demonstrated sandwich-type CL immunoassay based on carboxyl-functionalized MNPs modified with anti-α-humanchorionic gonadotropin (hCG) antibody [19]. Under optimized conditions, the assay was sensitive to 1.184 ng mL^−1^ analyte within the dynamic range of 2 to 100 ng mL^−1^. The proposed method could be easily adapted for hCG quantitative detection in serum samples by utilizing the potent advantages of the bioassay for clinical diagnosis (decrease in immune-reagents dosage, high-sensitivity, rapid and simplified protocol) [19].

**Fig. 1:**
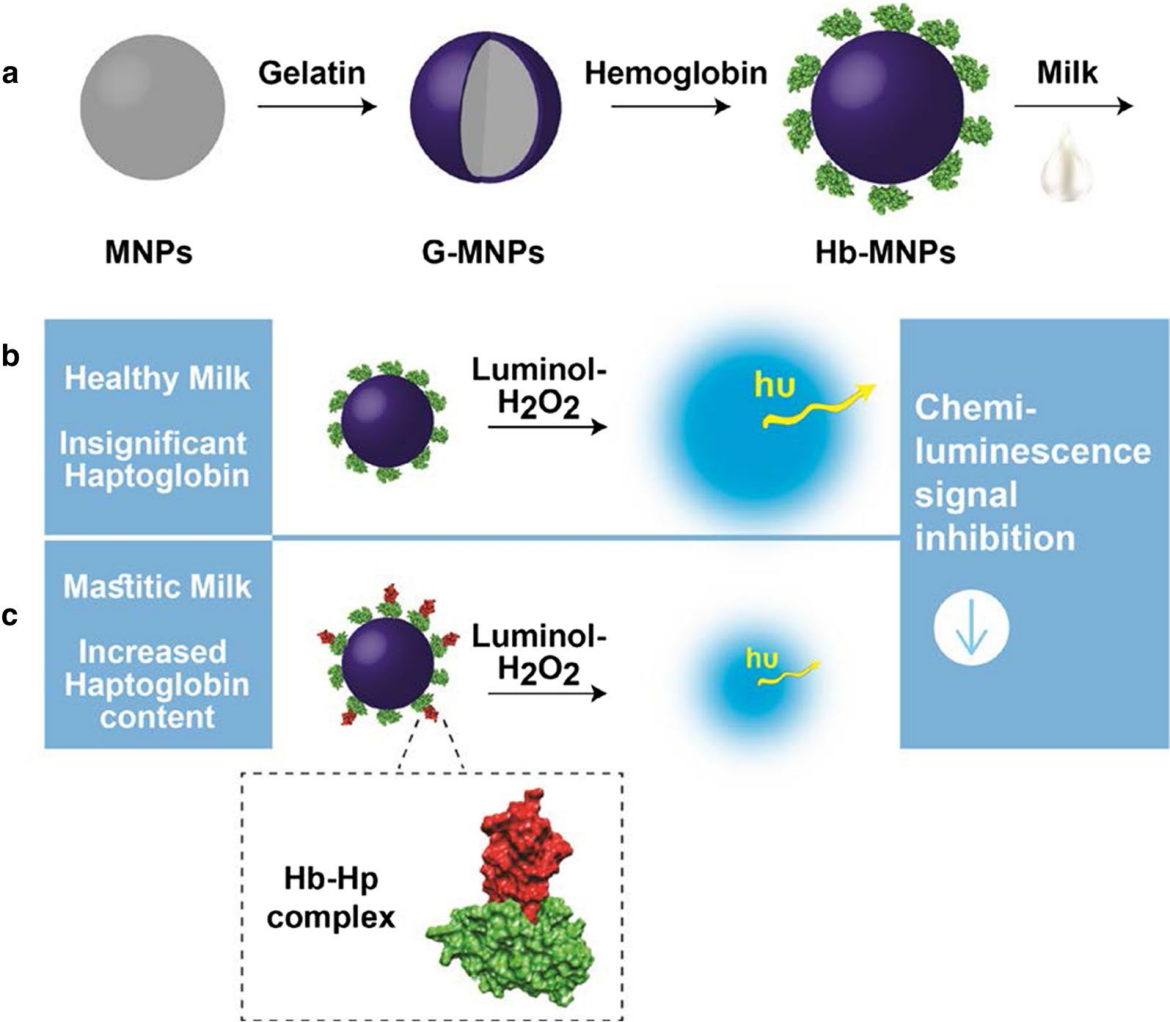
Schematic illustration of the CL bioassay concept for Hp detection. **a** MNPs were modified with gelatin (G-MNPs) followed by Hb bioconjugation onto the solid support (Hb-MNPs). The resulting nanoparticles were applied onto different milk qualities and separated through magnetic field attraction before CL analysis. The CL signal is inversely correlated to the inherent Hp content presenting **b** increased CL values of the healthy milk sample due to insignificant Hp content and **c** low CL emission of mastitic milk sample containing increased Hp concentrations

Herein, we have designed and fabricated CL bioassay for ultrasensitive analysis of Hp content in bovine milk, by employing the specific interaction of Hb-modified MNPs. The resulting Hb-Hp complex formation inhibit the peroxidase-like activity of luminol/H_2_O_2_-Hb-MNPs system, thus offering means for analytical evaluation of Hp content, as schematically illustrated in Fig. 1 [9, 10]. The correlation between CL signal and Hp concentration of dissimilar milk samples was effectively attained. Moreover, the impact of SCC levels and pathogen types, i.e., *Streptococcus dysgalactiae* and *Escherichia coli* (*Strep. dysgalactiae* and *E. coli*, respectively), on the secreted Hp was studied in comparison to healthy udder. Finally, the bioassay performances were compared with the sensing data of a commercial ELISA kit.

## Materials and methods

### Materials

Iron(III) chloride hexahydrate, iron(II) chloride tetrahydrate, ammonium hydroxide, analytical grade buffers, bovine Hp, bovine Hb, glutaric dialdehyde (50 wt%), gelatin, H_2_O_2_, luminol sodium salt and sodium hydroxide were obtained from Sigma-Aldrich Chemicals. Hp ELISA Kit was supplied by Abcam (cat. num. ab137977). Milli-Q water (18 MΩ cm) was used for all the denoted experiments.

### MNPs synthesis

Iron(III) chloride hexahydrate (240 mg, 0.9 mmol) dissolved in deoxygenated milliQ purified H_2_O (4.5 mL) was mixed with an aqueous solution of iron(II) chloride tetrahydrate (97.5 mg, 0.45 mmol, 4.5 mL H_2_O). This solution was kept under nitrogen atmosphere and ultra-sonicated (Bransonic^®^ ultrasonic cleaner bath, 2510E MTH model, 42 kHz at full power) for 5–10 min at room temperature (RT). Then, a concentrated 24% weight ammonium hydroxide aqueous solution (0.75 mL) was introduced in one shot, resulting in immediate black precipitation of magnetite (Fe_3_O_4_) nanoparticles (MNPs). Sonication was continued for 10 additional minutes. Resulting MNPs were magnetically decanted (using a strong external magnet) and washed 3 times with ultrapure water (30 mL) until neutrality. Then, brilliant black free flowing MNPs were stored for 2 h as a 30 mL suspension in ultrapure water before any further processing (an aging process), washed once again and redispersed in 25 mL of ultrapure water.

### Instrumentation

The absorption spectra and CL intensities were analyzed with Varioskan™ LUX (by Thermo Scientific, Waltham, MA, USA), a multimode microplate reader [9]. The structural morphology and size distribution of the different nanoparticles were studied by transmission electron microscopy (TEM), JEOL JEM-1400, operated at 120 kV. Dynamic light scattering (DLS) was used to analyze the hydrodynamic diameter of the functionalized MNPs using a Zetasizer Nano ZS (Malvern Instruments Ltd., Malvern, UK) while performing the measurements at RT [9]. The results were obtained from number-based distributions. Attenuated total reflectance Fourier transform infrared (ATR-FTIR) spectroscopy was used to further confirm MNPs surface modification using Thermo Scientific Nicolet iS50, as previously noted [27]. Image analysis was performed by ImageJ software.

### Milk samples collection and preparation

Milk samples were obtained from specific quarters of Holstein cows (Volcani Center) depicting healthy, spontaneous sub-clinical or clinical mastitis, as previously described [10]. All milk samples prior optical examination were defatted by mild centrifugal force of 2000×*g* for 10 min and tenfold diluted [10].

### MNPs biofunctionalization

MNPs biofunctionalization was performed following a reported protocol by Nirala et al. (Fig. 1a) [10]. Briefly, 200 µL gelatin aqueous (1% wt/v) solution were added onto 1 mL of diluted MNPs (0.49 mg mL^−1^) and allowed to react for 1 h at RT during mild orbital shaking (750 rpm). The resulting solution was thoroughly cleaned with ultrapure water (1 mL) by collecting the modified nanoparticles with a homemade magnetic separation unit (using two neodymium cubes 15 × 15 × 15 mm, N35, Ni-Cu-Ni coating, 8.5 kg holding force each) for 5 min and redispersing the content to a final volume 1 mL (repeated three times) resulting in gelatin functionalized MNPs (G-MNPs). Next, G-MNPs were incubated with 200 µL of 2.5 wt% glutaraldehyde solution for 0.5 h at RT to activate functional binding residues followed by repeating washing procedure to a final volume of 1 mL. Finally, 200 µL of Hb solution (1, 10 and 100 µg mL^−1^) in ultrapure water was allowed to crosslink for 1 h at RT, proceeded by a post-cleaning procedure to a final volume of 0.5 mL, resulting in Hb-modified MNPs (Hb-MNPs). The immobilized Hb content was analyzed indirectly by quantifying the residual washing solution content by Bradford dye-binding method [28].

### Bioassay calibration and Hp quantification in milk

Reference bovine Hp solutions (different concentrations in the range of 0 to 1 µg mL^−1^ in PBS pH 7.4) were reacted with Hb-MNPs for 30 min at RT during mild shaking (200 and 500 µL, respectively), followed by the above post-cleaning procedure to exclude any unbounded or interfering species. Next, 200 µL of tenfold diluted healthy milk samples (containing insignificant Hp residues) were added onto the solution, allowed to react for 30 min at RT and cleaned similarly. The resulting solution (100 µL) was calibrated by instantaneous CL examination following the addition of 100 µL of alkaline luminol/H_2_O_2_ solution (1.5 mM sodium hydroxide, 1 µM H_2_O_2_, 45 µM luminol sodium salt) while evaluating the emitted radiation values [10]. Milk samples were analyzed similarly, i.e., 200 µL tenfold diluted of unknown samples were incubated for 30 min with Hb-MNPs at RT during mild shaking, followed by a post-cleaning in ultrapure water. CL studies of the dissimilar milk qualities were evaluated immediately by applying alkaline luminol/H_2_O_2_ solution. The obtained emission values were used to calculate Hp concentrations based on the calibration curve and were paired to the values of bovine Hp ELISA kit [10].

### Statistical analysis

T-test with a minimum confidence level of 0.05 was used for statistical significance (assuming unequal sample sizes and unequal variance). All values are reported as the mean ± SD (n ≥ 3).

## Results and discussion

### Optical and structural characterization of biofunctionalized MNPs

**Fig. 2:**
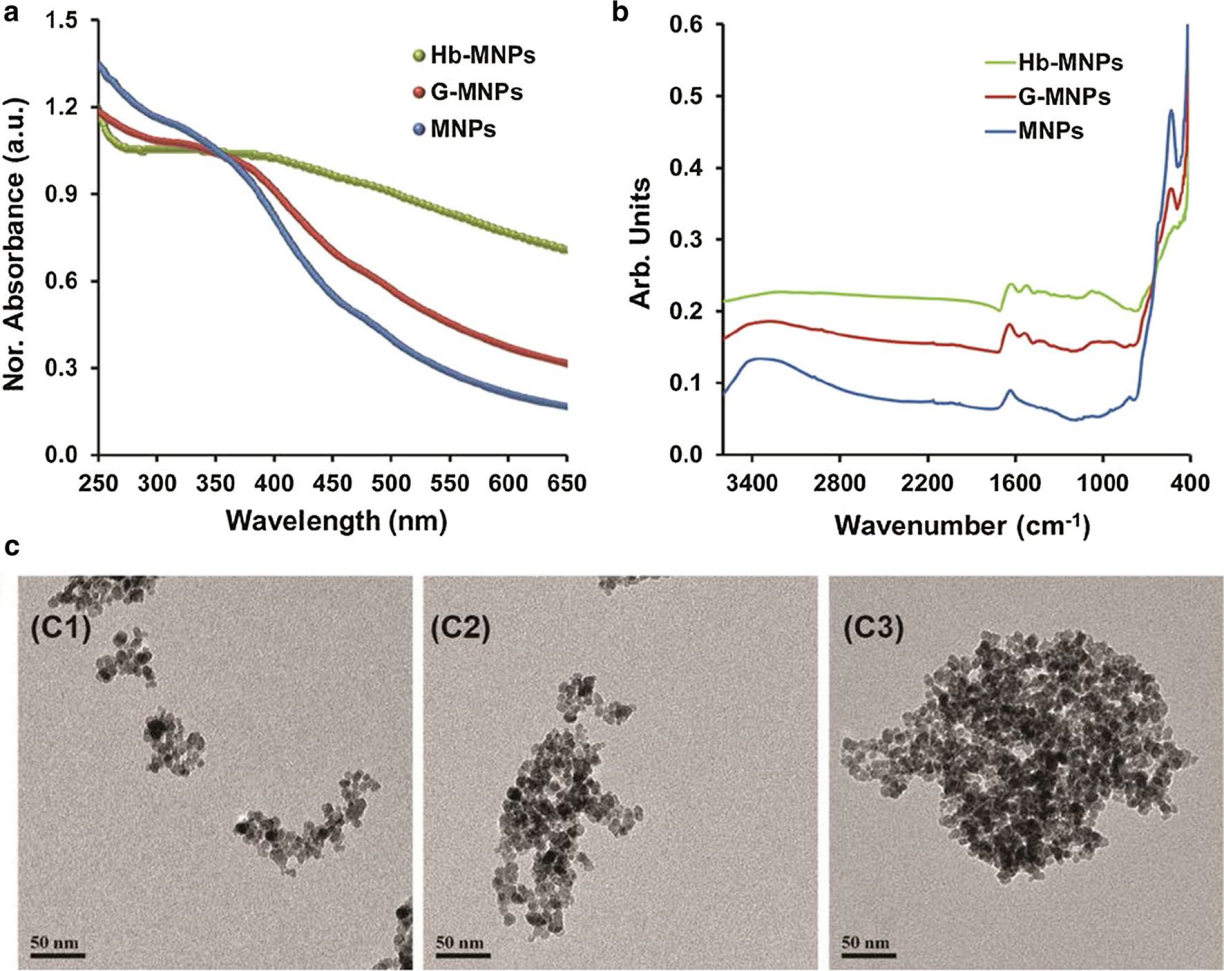
**a** Normalized UV–VIS and **b** ATR-FTIR spectra of the modified MNPs: bare MNPs; G-MNPs and Hb-MNPs; **c** TEM images of the modified MNPs: (c1) bare MNPs, (c2) G-MNPs and (c3) Hb-MNPs presenting aggregates of 96 ± 31, 175 ± 39 and 310 ± 121, respectively. Data are reported as mean ± SD (n ≥ 8) obtained by ImageJ software

The overall concept of the presented CL bioassay is to utilize the binding capacity and the catalytic activity of bovine Hb-modified carriers. Thus, Hb was applied on magnetic Fe_3_O_4_ nanoparticles through a standard biofunctionalization approach [10]. Briefly, MNPs were synthesized by using bath sonication method by mixing iron(III) chloride and iron(II) chloride solutions according to a previous work [29]. The obtained MNPs were subsequently decorated by gelatin molecules for attributing functional groups for cross-linking Hb on the exterior of the magnetic carriers and for minimizing the non-specific adsorption of milk constituents during the bioassay protocol [9, 10]. Next, the gelatin-free amino groups were modified with glutaraldehyde to activate the surface for Hb immobilization [30]. Note: assuming insignificant particles loses throughout the vigorous rinsing steps and biofunctionalization processes the concentration was set to 0.49 mg/mL of magnetite. The resulting MNPs surface modifications and morphologies were characterized by UV–VIS, ATR-FTIR and TEM, see Fig. 2. The absorbance spectra show a characteristic shoulder band edge that is red shifted, i.e., 363, 371, 396 nm for bare MNPs, G-MNPs, Hb-MNPs, respectively (Fig. 2a). The red shift is mainly ascribed to the nanoparticles collective diameter increase upon surface loading with gelatin and subsequently cross-linking with Hb creating cluster formation. MNPs surface characterization was further examined by ATR-FTIR studies, see Fig. 2b. The bare MNPs IR spectrum depicts intense characteristic Fe–O stretching vibration peak at 532 cm^−1^ of bulk magnetite, absorption peak at 1634 cm^−1^ denoted to hydroxyl groups bending vibration, and a broad peak around 3347 cm^−1^ accredited to the stretching vibration of –OH residues in aqueous media [25]. Gelatin modification is characterized by the addition of two new peaks at 1636 and 1539 cm^−1^ corresponding to amide I and amide II of the protein, respectively, while the intensity of Fe–O stretching vibration is decreased [20, 24]. Lastly, similar spectrum was obtained for Hb-MNPs, while further decrease in the Fe–O peak intensity is shown while both amide I and amide II peaks intensities are respectively increased as additional protein residues are attached onto the G-MNPs surface. Overall, the additional absorption peaks of G-MNPs and Hb-MNPs spectra suggest outer protein coverage. Additionally, Additional file 1: Table S1 presents the peak area ratio of both amide bonds *vs* the peak area of Fe–O, which is increased upon surface modification. Finally, the fine structures of the different MNPs were elucidated by TEM, see Fig. 2c. Figure 2 (c1) shows representative bare MNPs which are relatively uniform in size and are within the range of 9.8 ± 0.5 nm as individual nanoparticles and mini aggregates of 96 ± 31 nm in size. The unmodified nanoparticles upon protein adsorption and cross-linking are size augmented and form greater clusters of 175 ± 39 and 310 ± 121 nm for G-MNPs and Hb-MNPs, respectively, see Fig. 2 (c2) and (c3). Additional file 1: Table S2 depicts the collective hydrodynamic cluster dimensions acquired by DLS measurements. The obtained results are in agreement with UV–VIS and TEM studies indicating supercluster formation upon sequential surface modification (211 ± 6, 469 ± 31 and 535 ± 173 nm for bare MNPs, G-MNPs and Hb-MNPs, respectively). It should be noted that the DLS data overestimation with respect to TEM dimensions, is attributed to the different factors affecting intrinsic measurements, as previously shown [31–33].

### CL characterization of modified MNPs

**Fig. 3:**
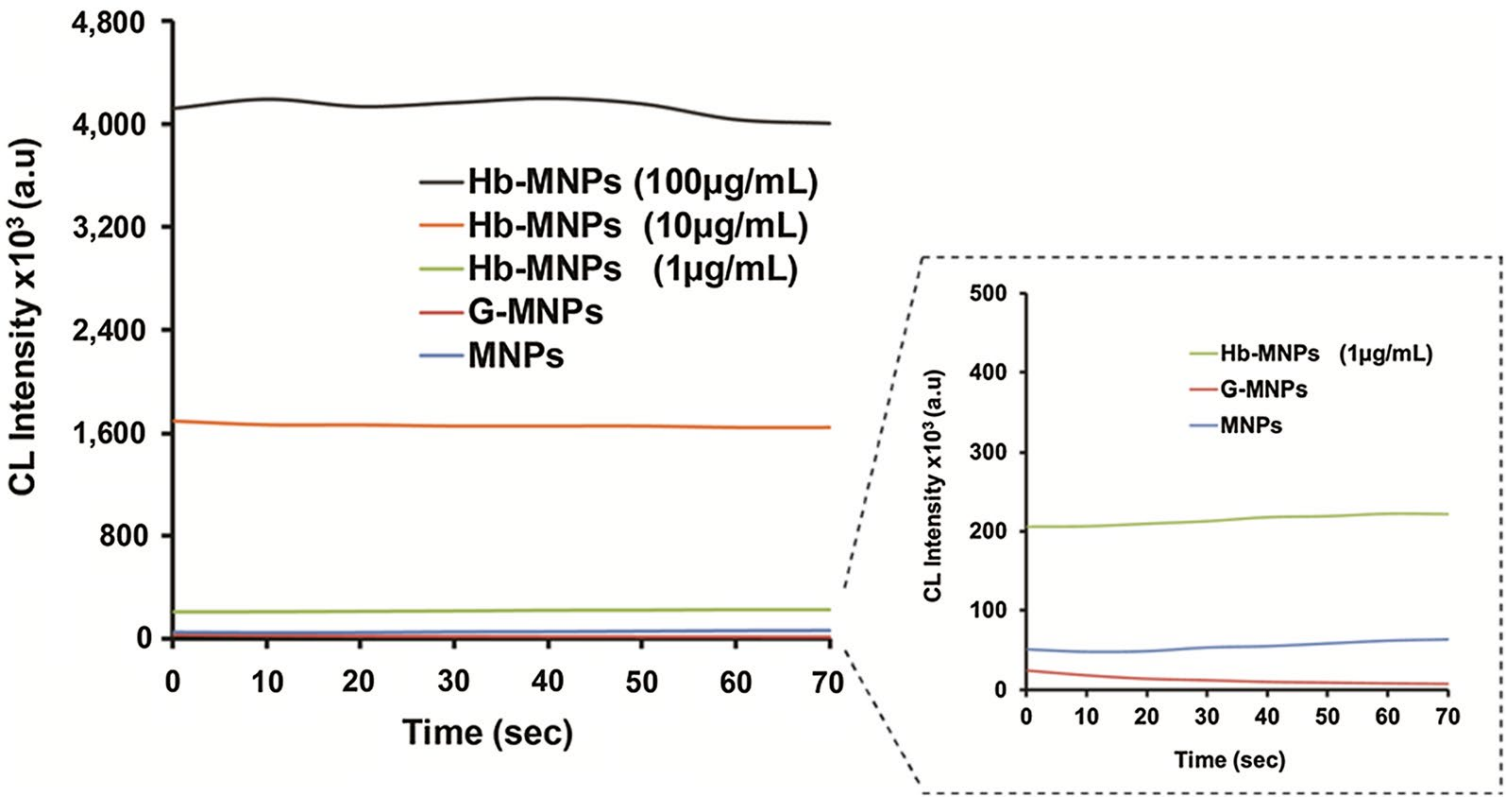
CL signal stability of the different MNPs modifications: bare MNPs; G-MNPs and Hb-MNPs, over time for the luminol system. The latter modification was assessed for three concentrations of catalyst: 1, 10, and 100 µg mL^−1^ of Hb. Inset: enlargement of the different MNPs modifications

The peroxidase mimetic activity of Hb in luminol/H_2_O_2_ CL system is well documented presenting the applicability of the sensing concept for ample analytical detection approaches [17, 34–38]. Therefore, the incorporation of a catalyst onto magnetic support via surface chemistry should eliminate any opto-chemical response that can be translated to intensified background luminescence. Meaning, the applied counterparts of the carrier system (gelatin, cross-linker and the magnetic carrier itself) should eliminate any false-positive emitted radiation, ensuring optically inert substances. Thus, the kinetic CL signal stability of the different MNPs modifications within the luminol system were investigated. Figure 3 depicts the obtained CL values of unmodified and protein (i.e., gelatin and Hb)-decorated magnetic carriers. Indeed, extensively stable CL signals of Hb-MNPs at various potential concentrations of catalyst were perceived (4122 ± 71 × 10^3^, 1661 ± 15 × 10^3^, 214 ± 7 × 10^3^ a.u. for 100, 10, 1 µg mL^−1^, respectively) under optimized experimental conditions [10]. It should be noted that although MNPs were functionalized with high content of Hb (6.4 ± 1.4, 0.6 ± 0.4 µg Hb per 0.49 mg magnetite for 100, 10 µg mL^−1^, respectively, see Additional file 1: Table S3), the optical response retained its stability over the entire duration of the assay, thus presenting controlled consumption of the reaction constituents. In the absence of catalytically active biomolecules, the reactions of both bare MNPs and G-MNPs with luminol/H_2_O_2_ system showed weak CL emission (54 ± 6 × 10^3^ and 15 ± 6 × 10^3^ a.u., respectively). Moreover, the different Hb concentrations on the exterior of MNPs carriers were examined within two dissimilar milk qualities (healthy and clinical mastitis) with respect to milk free conditions. Additional file 1: Figure S1 presents that the boundary conditions for quality estimation, low and high Hp concentrations, could be significantly differentiated by utilizing the highest catalyst content (100 µg mL^−1^ of Hb) for the noted bioassay. Considering the pronounced CL signal, stability over time and the ability to differentiate dissimilar milk qualities, our previously reported optimized conditions (alkaline luminol/H_2_O_2_ solution) were adapted for the analytical evaluation of Hp within real milk samples [9, 10], while modifying the magnetic carrier with a substantial content of Hb (100 µg mL^−1^).

### CL bioassay within milk samples

**Fig. 4:**
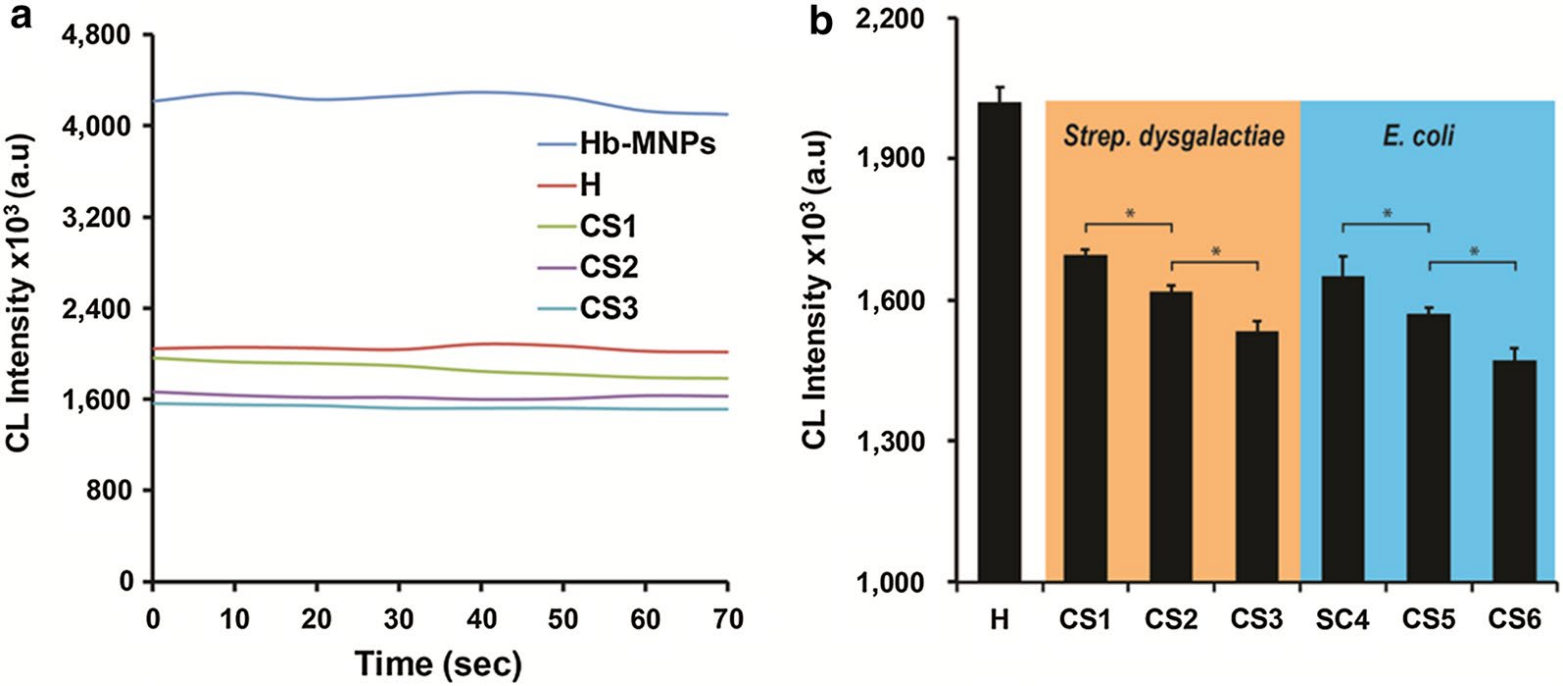
**a** CL signal stability of the bioassay within dissimilar milk samples qualities (healthy, sub-clinical or clinical mastitis) and Hb-MNPs (control); **b** The corresponding CL values within different milk samples for two predominant pathogens (*Strep. dysgalactiae* and *E. coli*). Data are reported as mean ± SD (n ≥ 3). *Statistically different (t-test, p < 0.05)

The practical aspect and analytical determination of the developed bioassay for Hp detection within real samples was investigated for comprehensive biomarker content evaluation. Table 1 summarizes the analyzed milk samples acquired from various udders, which represent the entire milk quality spectrum. Three levels of SCC, approx. 300,000, 600,000 and 1,000,000 cells mL^−1^ for sub-clinical and clinical mastitis, positive with a specific mastitis causative pathogen (e.g., *Strep. dysgalactiae* and *E. coli*) were studied. Healthy milk (sample H) was used as a negative control as no traces of any photogenic contaminants were obtained through microbiological examination and low SCC score of 71,000 cells mL^−1^ were acquired, thus expecting minute Hp concentrations. It should be noted that sample H was accepted as a representative of other healthy milk samples with similar negative biological contamination and insignificant SCC values (< 100,000 cells mL^−1^) for this study. Defatted milk samples were reacted with Hb-MNPs according to the noted procedure using optimized conditions. It is expected that upon acute-phase response of cows suffering from mastitis will induce intensified Hp concentrations both in milk and plasma in proportion to the clinical severity of the occurring inflammation. Increased Hp levels will profoundly induce protein–protein (Hp-Hb) complexes formation, thus hindering the peroxidase-like activity of Hb-MNPs within luminol CL system and producing reduced CL intensities. Figure 4a depicts CL signal stability of the bioassay within different milk samples qualities (healthy, sub-clinical or clinical mastitis) in comparison to a milk-free solution (only Hb-MNPs), expecting significant CL values and stability of the latter. Indeed, the CL values were reduced upon deterioration of milk quality (escalation in SCC levels), presenting values of 1871 ± 65 × 10^3^, 1626 ± 22 × 10^3^, 1535 ± 16 × 10^3^ a.u. for CS1, CS2, CS3 (all positive with *Strep. dysgalactiae*) in comparison to the negative control (sample H with CL values of 2052 ± 24 × 10^3^ a.u.). It should be noted that all examined milk samples presented similar CL signal stability throughout the entire duration of the bioassay in comparison to the stability of Hb-MNPs. Figure 4b presents the corresponding CL values within different milk samples qualities by comparing two predominant pathogens of mastitis (*Strep. dysgalactiae* and *E. coli*). Similar CL output was shown for samples CS4, CS5, CS6 (all positive with *E. coli* pathogen). The resulting further confirm the correlation between the emitted radiation of the presented CL system and the inherent Hp concentration within milk. Samples CS3 and CS6 depict low CL values of 1534 ± 22 × 10^3^ and 1458 ± 38 × 10^3^ a.u., respectively. The inhibited catalytic activity of Hb-MNPs within these samples can be attributed to increased biomarker concentrations due to acute clinical state of the examined cows, both positive with pathogenic microbial contamination and elevated SCC scores, above 1000,000 cells mL^−1^. Overall, these results efficiently differentiate between the different milk qualities based on the proposed liquid phase CL system and offer feasible means for deducing the severity of the occurring bovine mastitis. Moreover, the intensified CL values of highly-loaded catalyst content on the magnetic carrier combined with the option to pre-concentrate the attached target analyte are highly important for on-site detection application, in which less sophisticated or sensitive devices can be used for routine mastitis severity evaluation.

**Table 1:** SCC values and causative pathogen of the obtained milk samples

Sample	SCC (cells mL^−1^)	Pathogen
H	71,000	N/A
CS1	353,000	*Strep. dysgalactiae*
CS2	495,000	*Strep. dysgalactiae*
CS3	> 1,000,000	*Strep. dysgalactiae*
CS4	300,000	*E. coli*
CS5	636,000	*E. coli*
CS6	> 1,000,000	*E. coli*

Healthy milk sample (H), cattle sick milk sample (CS)

### Milk Hp quantification based on CL bioassay

**Fig. 5:**
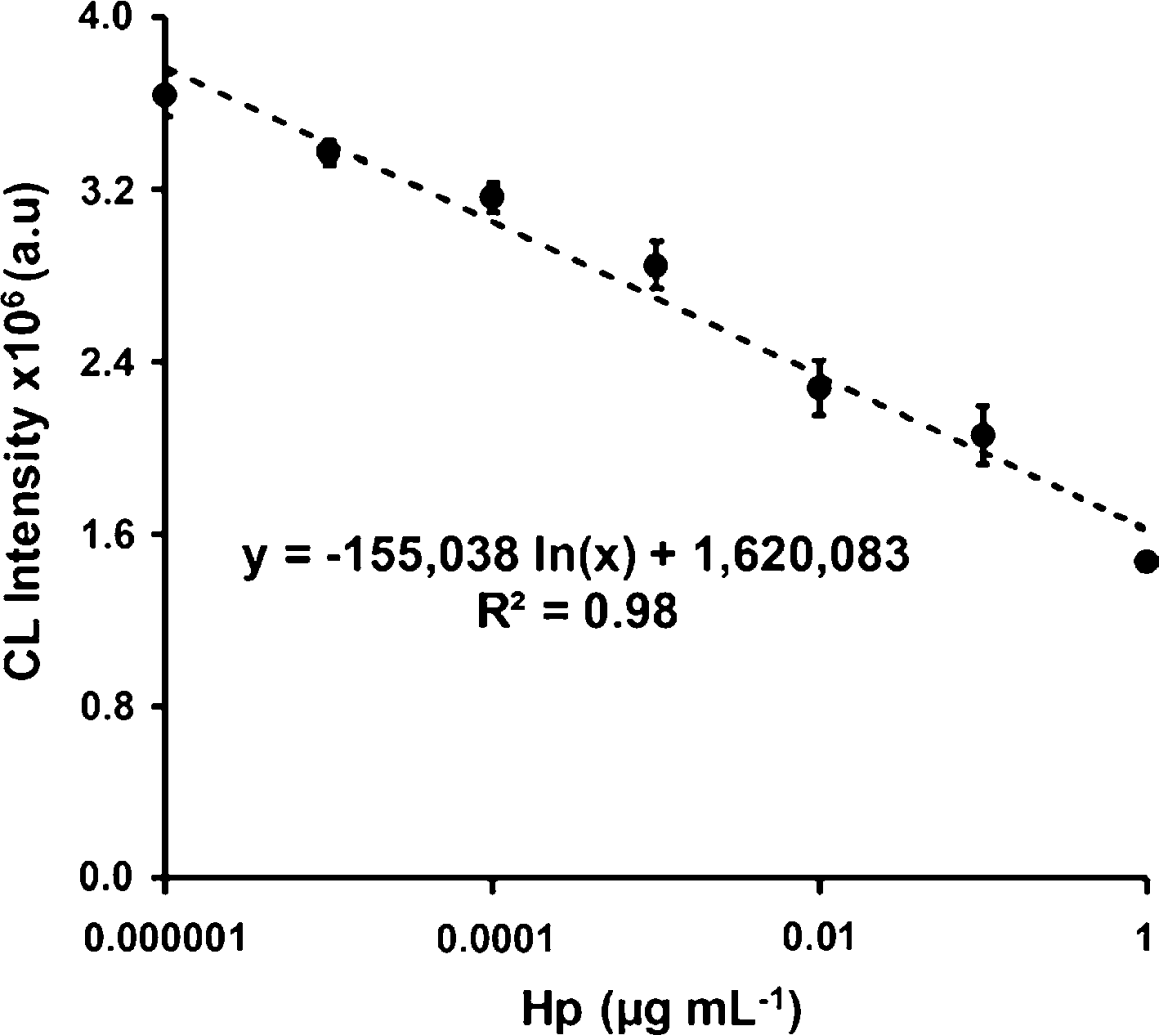
Calibration curve of the CL bioassay for standard Hp concentrations based on Hb-MNPs. Data are reported as mean ± SD (n ≥ 3)

The CL bioassay was calibrated within the dynamic range of 1 pg mL^−1^ to 1 µg mL^−1^ Hp, linking the optical output *vs* the biomarker concentration. Corresponding to the obtained CL data, profound Hp degree will hinder the catalytic response ascribed to protein–protein (Hb-Hp) composite association. Figure 5 presents the calibration curve of the CL bioassay for standard Hp concentrations based on Hb-MNPs sensing concept acquired under optimized conditions. Indeed, proportional CL decrease within the entire dynamic range was shown for higher target analyte concentrations, while presenting the fitted regression of the calibration curve CL = − 155,038 Ln (C_Hp_) + 1,620,083 (*R*^2^ = 0.98). The limit of detection (LOD) of 0.89 pg mL^−1^ was calculated using the equation of $y_{b} - 3std_{b}$, where $y_{b}$ is the averaged CL intensity measured for maximal CL intensity without any Hp residuals and $3std_{b}$ is the associated standard of deviation, as previously shown [39]. The presented LOD is lower than the many reported optically based research studies (e.g., absorbance, fluorescence, CL, and surface plasmon resonance) utilizing both photon count sensitivity of the CL concept and the pre-concentration of highly loaded catalyst on the magnetic carriers [23, 40–43]. It should be noted that a healthy milk sample (containing insignificant Hp residues) was added to each Hp reference condition to simulate interferences from milk constituents. Additional file 1: Figure S2 depicts a representative CL stability and averaged data of the healthy milk, which suggests Hp content below the dynamic range of the calibration curve due to elevated emission values. The latter was further verified by calibrating the CL system in buffer conditions presenting similar CL decrease with respect to Hb catalytic inhibition, see Additional file 1: Figure S3. An alternative means to simulate milk constituents’ interferences is by depleting Hp from the milk by specific interaction with Hb-MNPs followed by magnetic separation and sequentially using the Hp-free milk as a blocking simulant [44]. Next, the regression equation was used for assessing Hp content within the studied samples, see Table 2. The calculated Hp values support the CL intensities study of the various milk qualities, presenting a similar relationship to the clinical severity of the occurring inflammation. Both pathogens induce similar inflammatory response of increased Hp concentrations at higher SCC values. However, the acute-phase response of *E. coli* contamination over *Strep. dysgalactiae* was pronounced by secreting higher Hp content onto milk while comparing similar SCC values (CS1, CS2, CS3 vs. CS4, CS5, CS6, respectively). The causative pathogen origin has been previously demonstrated in several field studies that the animal’s immune response is triggered or influenced differently while comparing clinical stage mastitis, favoring *E. coli* contamination over *Strep. dysgalactiae*. It should be noted that *E. coli* cause the inflammation around parturition and during early lactation, however trigger severe systemic clinical symptoms at all inflammatory stages [45]. Lastly, the performance of CL bioassay was compared with bovine ELISA technique to verify the validity and compliance of Hp analysis in real milk samples to the standardize technique used to differentiate milk qualities. Indeed, similar Hp values trends were obtained for all analyzed samples at various clinical stages of the occurring mastitis, considering the SCC scores and the causative bacteria. The Hp concentrations based on Hb-MNPs overestimate the biomarker content within milk than the equivalent ELISA results, which can be ascribed to the sensitivity differences between the optical techniques. The CL bioassay overpowers the conventional assay by measuring photon count rather than colorimetric absorbance, thus presenting lower LOD values [10, 21, 46]. To further elucidate the calculated Hp differences, a control experiment with known Hp content (0.005 µg mL^−1^) in buffer and in milk was applied to both sensing techniques (Additional file 1: Figure S2). Additional file 1: Table S4 summarizes the calculated Hp concentrations using Hb-MNPs CL emission based on calibration curve in buffer and in milk conditions and bovine ELISA. Similarly to the performances in real milk samples, the CL bioassay examined in buffer conditions overestimates the spiked Hp concentration, while the ELISA quantification underestimates its content. The recovery values of both techniques are 120% and 92% for CL bioassay and ELISA, respectively, while methods deviation is 130%. The recovery values of the latter condition (milk) are augmented for both techniques and the methods deviation is 133%. The deviation can be attributed to the wide range of reference Hp concentrations, which are susceptible to heteroscedasticity within semi-logarithmic scale. Moreover, the absolute analyte levels measured by commercial ELISA are known to vary between different manufacturers using similar standard solutions and prone to samples’ constituents false interactions [47, 48]. Herein, the concentration trend in complex media are paramount rather the obtained absolute values which are used to determine relative inflammation differences or alternatively to follow the therapy efficiency. Despite the concentration deviation both in milk and in buffer conditions, the presented assay can precisely differentiate dissimilar milk qualities and indicate on animals’ clinical stage. The intensified CL signal is advantageous in terms of flexibility and applicability for on-field detection, reducing both the protocol duration and costs per assay. The presented assay can be easily adapted for other protein classes inspection upon minor assay modification and improvement. Overall, the developed CL bioassay based on magnetic carriers, which specifically target and pre-concentrate the molecule of interest, is suitable for Hp analysis in milk samples.

**Table 2:** Hp concentrations in milk samples using Hb-MNPs CL emission system in comparison to bovine ELISA

Sample	Hp Hb-MNPS (µg mL^−1^)	Hp ELISA (µg mL^−1^)
H	0.8 ± 0.2	0.7 ± 0.1
CS1^a^	6.1 ± 0.4	4.7 ± 0.3
CS2^a^	10.1 ± 0.9	6.0 ± 0.4
CS3^a^	17.6 ± 2.5	10.6 ± 0.9
CS4^b^	8.4 ± 2.4	6.0 ± 0.8
CS5^b^	13.8 ± 1.2	9.1 ± 0.9
CS6^b^	26.3 ± 4.3	17.7 ± 0.4

Healthy milk sample (H), Cattel sick milk sample (CS)

Data are reported as mean ± SD (n ≥ 3)

^a^Positive with *Strep. dysgalactiae* pathogen

^b^Positive with *E. coli* pathogen

## Conclusions

An ultrasensitive Hp biomarker detection was designed based on the binding capacity of Hb-modified MNPs, while assessing their catalytic activity inhibition within the CL bioassay. Hp concentrations in real milk samples were increased in correlation to the severity of the occurring mastitis and SCC levels. The main advantage of the presented sensing concept is the ability to detect Hp at clinically relevant concentrations in real milk samples for early bio-diagnostic detection of mastitis, using a simple and cost-effective experimental setup. Moreover, the study demonstrates the possibility to design a simple yet reliable sensing platform to target predictive inflammation biomarkers, which are secreted into plasma or milk at acute-phase response, to differentiate the clinical stage of the animal (sub-clinical, clinical or chronical) and thus adjusting the precise treatment. Furthermore, this proof-of-concept can be easily adapted for any human disease associated with Hp (i.e., diabetes, neurological and cardiovascular disorders).

## Supplementary information

**Additional file 1: Table S1.** Peak area comparison of amide bonds vs. Fe–O for the different MNPs modifications obtained by ATR-FTIR. **Table S2.** MNPs size distribution obtained by DLS. **Table S3.** Hb binding efficiency to MNPs surface. **Table S4.** Hp concentrations in spiked buffer and milk samples using Hb-MNPs CL emission system in comparison to bovine ELISA. **Figure S1.** CL values of the magnetic bioassay for three concentrations of Hb catalyst (1, 10, and 100 µg mL^−1^) within dissimilar milk samples qualities. **Figure S2.** CL signal stability of the Hb-MNPs bioassay within healthy milk sample and spiked Hp in buffer and milk. **Figure S3.** Calibration curve of the CL bioassay for standard Hp concentrations based on Hb-MNPs in buffer.

## Data Availability

All data generated or analyzed during this study are included in this published article.
