# Supplementary material for: Ultrasensitive haptoglobin biomarker detection based on amplified chemiluminescence of magnetite nanoparticles
Source: J Nanobiotechnology. 2020 Jan 7;18:6. doi: 10.1186/s12951-019-0569-9 (PMC6945394; doi:10.1186/s12951-019-0569-9)
Supplement: Supplementary file 1 — Additional file 1: Table S1. Peak area comparison of amide bonds vs. Fe–O for the different MNPs modifications obtained by ATR-FTIR. Table S2. MNPs size distribution obtained by DLS. Table S3. Hb binding efficiency to MNPs surface. Table S4. Hp concentrations in spiked buffer and milk samples using Hb-MNPs CL emission system in comparison to bovine ELISA. Figure S1. CL values of the magnetic bioassay for three concentrations of Hb catalyst (1, 10, and 100 µg mL−1) within dissimilar milk samples qualities. Figure S2. CL signal stability of the Hb-MNPs bioassay within healthy milk sample and spiked Hp in buffer and milk. Figure S3. Calibration curve of the CL bioassay for standard Hp concentrations based on Hb-MNPs in buffer. [file 12951_2019_569_MOESM1_ESM.docx]

**Additional information**

**Ultrasensitive haptoglobin biomarker detection based on amplified chemiluminescence of magnetite nanoparticles**

*Narsingh R. Nirala^1^, Yifat Harel^2^, Jean-Paul Lellouche^2^, Giorgi Shtenberg^1*^*

^1^ Institute of Agricultural Engineering, ARO, the Volcani Center, Bet Dagan 50250, Israel

^2^ Department of Chemistry, Nanomaterials Research Center, Institute of Nanotechnology & Advanced Materials (BINA), Bar-Ilan University, Ramat-Gan 5290002, Israel

*Corresponding author: [giorgi@agri.gov.il](mailto:giorgi@agri.gov.il)

**Table S1.** Peak area comparison of amide bonds *vs.* Fe-O for the different MNPs modifications obtained by ATR-FTIR

| Sample | Peak Area | | Ratio ($\frac{\upsilon_{(amide I and II)}}{\upsilon_{(Fe-O)}}$) |
| --- | --- | --- | --- |
|  | υ_(amide I and II)_^a^ | υ_(Fe-O)_^b^ |  |
| MNPs | 2.16 | 6.17 | 0.35 |
| G-MNPs | 3.92 | 2.33 | 1.68 |
| Hb-MNPs | 3.55 | 0.65 | 5.46 |

^a^ 1474-1709 cm^-1^.

^b^ 479-603 cm^-1^.

**Table S2.** MNPs size distribution obtained by DLS

| Sample |  | Averaged diameter  (nm) |
| --- | --- | --- |
| MNPs |  | 211±6 |
| G-MNPs |  | 469±31 |
| Hb-MNPs |  | 535±173 |

Data are reported as mean ± SD (n = 3).

**Table S3.** Hb binding efficiency to MNPs surface

| Hb  (µg mL^-1^) |  | Added Hb  (µg) | Washed Hb ^a^  (µg) | Immobilized Hb ^b^  (µg) |
| --- | --- | --- | --- | --- |
| 1 |  | 0.2 | N/A ^*^ | N/A |
| 10 |  | 2 | 1.4±0.4 | 0.6±0.4 |
| 100 |  | 20 | 13.6±1.4 | 6.4±1.4 |

^a^ Quantified by the Bradford dye-binding method.

^b^ Immobilized Hb content per 0.49 mg magnetite.

^*^ Below linear quantification range.


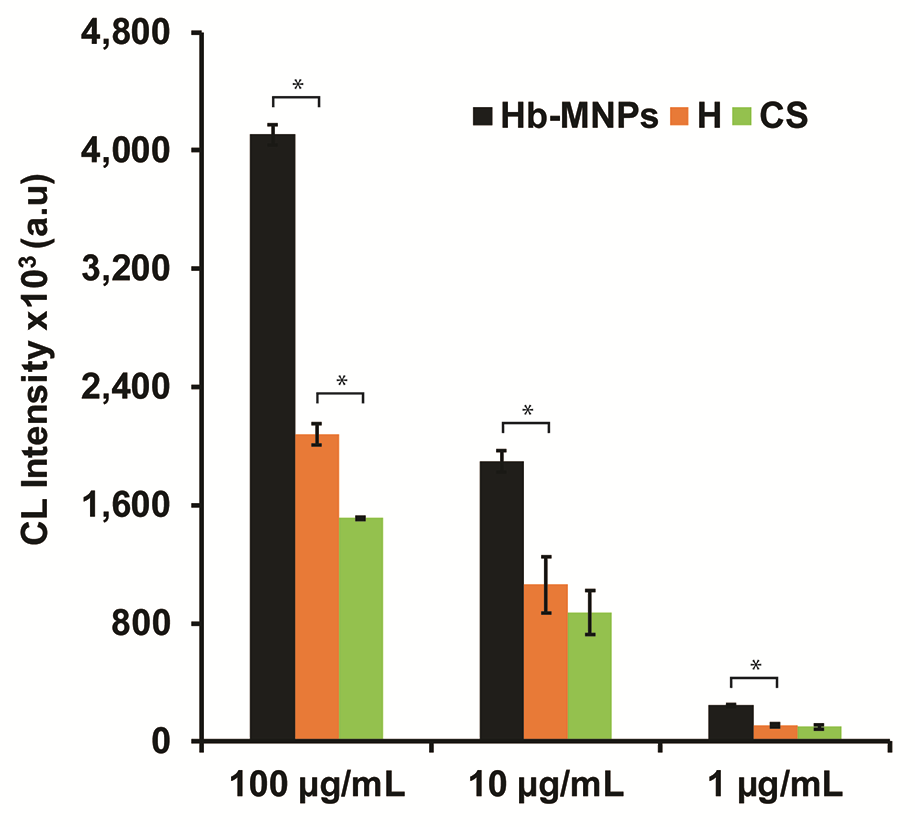


**Figure S1.** CL values of the magnetic bioassay for three concentrations of Hb catalyst (1, 10, and 100 µg mL^-1^) within dissimilar milk samples qualities: healthy (H), cattle sick (CS, representing clinical mastitis) and in buffer (Hb-MNPs used as a positive control). Data are reported as mean ± SD (n ≥ 3). * Statistically different (t-test, p<0.05).


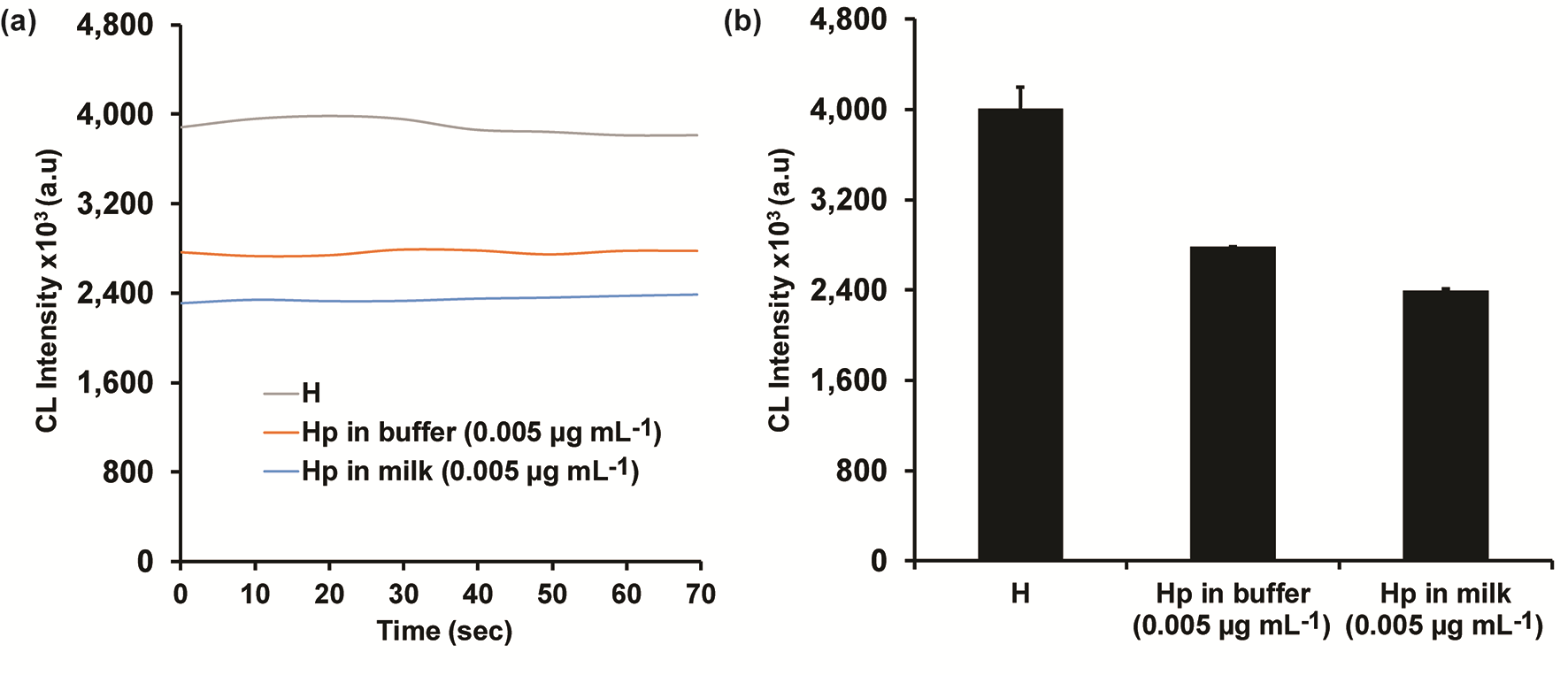


**Figure S2.** (a) CL signal stability of the Hb-MNPs bioassay within healthy milk sample (H) used for blocking (insignificant Hp content) and spiked Hp (0.005 µg mL^-1^) in buffer and milk, respectively; (b) The corresponding averaged CL data. Data are reported as mean ± SD (n ≥ 3).


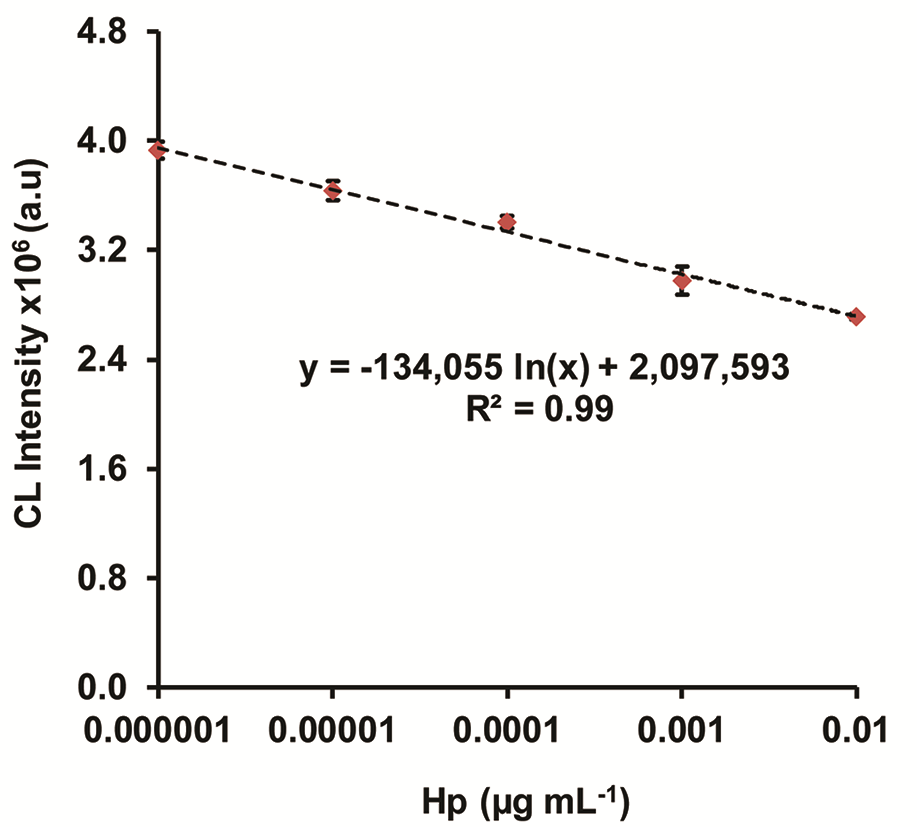


**Figure S3.** Calibration curve of the CL bioassay for standard Hp concentrations based on Hb-MNPs in buffer.

**Table S4.** Hp concentrations in spiked buffer and milk samples using Hb-MNPs CL emission system in comparison to bovine ELISA

| Spiked Hp  (µg mL^-1^) | Hp Hb-MNPS  (µg mL^-1^) | Hp ELISA  (µg mL^-1^) |  |  | CL  Recovery  (%) | ELISA  Recovery  (%) | Deviation  (%) ^b^ |
| --- | --- | --- | --- | --- | --- | --- | --- |
| 0.0050 in buffer | 0.0060±0.0002 ^a^ | 0.0046±0.0002 |  |  | 120 | 92 | 130 |
| 0.0050 in milk | 0.0069±0.0008 | 0.0052±0.0002 |  |  | 138 | 104 | 133 |

^a^ Calculated based on calibration curve in buffer conditions (milk-free assay, Figure S3).

^b^ Recovery of Hp output obtained by CL emission system with respect to bovine ELISA.
